# Quality of Life and Associated Factors among Patients with Schizophrenia Attending Follow-Up Treatment at Jimma Medical Center, Southwest Ethiopia: A Cross-Sectional Study

**DOI:** 10.1155/2020/4065082

**Published:** 2020-08-21

**Authors:** Defaru Desalegn, Shimelis Girma, Worknesh Tessema, Eyerusalem Yeshigeta, Teshome Kebeta

**Affiliations:** ^1^Department of Psychiatry, College of Health Science, Mettu University, Mettu, Ethiopia; ^2^Department of Psychiatry, Institute of Health, Jimma University, Jimma, Ethiopia; ^3^Department of Epidemiology, Faculty of Public Health, Jimma University, Jimma, Ethiopia

## Abstract

**Background:**

Schizophrenia is one of the most severe, chronic, and disabling mental disorders found globally. The chronic nature of the illness significantly interferes with functioning and results in a poor quality of life, but little is known about the quality of life among schizophrenia patients, in particular in low-income countries. Therefore, we assessed the quality of life and associated factors among patients with schizophrenia attending Jimma University Medical Center, Southwest Ethiopia.

**Methods:**

The hospital-based cross-sectional study design was employed to collect data from 352 study participants using a systematic random sampling technique from June to July 2018. Patients' sociodemographic characteristic, quality of life, psychopathology, medication adherence, comorbid physical illness, and substance use disorder were assessed. Data entry and analysis were done using EpiData version 3.1 and Statistical Package for the Social Sciences (SPSS) version 21.0, respectively. Variables with a *P* value < 0.05 in the final multiple regression models were declared to be associated with the outcome variable. *The Results*. The response rate of the study was 99.7%. The mean (±standard deviation) score of the World Health Organization Quality of Life Assessment Short Version Scale was 74.34 ± 15.83. Positive symptoms, negative symptoms, general psychopathologies, comorbid physical illness, khat use disorder, tobacco use disorder, and medication nonadherence were negatively associated with patient quality of life. However, monthly income was found to be positively associated with quality of life. *Conclusion and Recommendation*. The mean and standard deviation of the quality of life of people with schizophrenia is found to be 74.34 ± 15.83 in this study. The social relationship domain was found with the lowest mean score. Therefore, priority interventions need to be implemented to improve the social deficits.

## 1. Background

Schizophrenia is a severe and chronic mental disorder characterized by a group of symptoms that include distortions of thinking and perception, cognition and psychomotor abnormalities, avolition, and apathy as well as emotional and communication and emotional difficulties [1]. It affects general health, functioning, autonomy, and subjective well-being and alters individuals' perception of reality [2]. The lifetime prevalence of schizophrenia has generally been estimated at approximately 1% worldwide, and it is about the same in men and women [3]. The World Health Organization (WHO) estimated that about 24 million people suffer from schizophrenia globally [4].

In treating and managing schizophrenia, clinicians often focus on treating psychotic symptoms and ignore factors that are directly related to quality of life and prognosis of disease even though evaluation of patient's quality of life can help a lot in improving quality of care in patients with schizophrenia [5]. Thus, there was a shift in the concept of treatment with more emphasis on the aspect of the patient's quality of life [6]. Schizophrenia affects many areas of functioning; people with the illness often lead an isolated and a marginalized existence in poor housing, with a low income, little education, and poor vocational and social skills [2]. Most individuals are employed at a lower level, and the majority of them have limited social contacts outside of their family [7]. Patients with schizophrenia are also prone to stigma, which leads to discrimination and thus affects their life opportunities and quality of life [8].

Quality of life (QoL) in individuals with schizophrenia has been measured from both subjective and objective points of view [9]. However, the World Health Organization (WHO) focused on the subjective aspect and defines the quality of life as individuals' perception of their position in life in the context of the culture and value systems with their goals, expectations, standards, and concerns [10].

Compared to the general population, a study done in China found that patients with schizophrenia who were treated in primary care had a lower level of QoL [11]. A study done in Bangladesh found out that most of schizophrenic patients lead poor to moderate quality of life [12].

Factors that affect quality of life of schizophrenic patients include sociodemographic factors, type of psychopathology, severity of psychiatric symptoms, duration of untreated illness, duration of treatment [11, 12], social support, patients' educational status, patient income level, employment [13], and substance use disorder [14]. A study done on psychiatric symptoms and quality of life of patients with schizophrenia found that positive and negative symptoms were more strongly related to poor QoL, whereas general psychopathology showed a consistent negative relationship with QoL [15]. Another study found out that quality of life is negatively correlated with negative symptoms and general psychopathology [5]. A study done in Nigeria found that there were significant negative correlations between several domains of quality of life and medication adherence [16]. A study from Ethiopia found that sociodemographic factors like being divorced, having no formal education, and age and psychiatric symptoms were significantly associated with quality of life [17]. A better understanding of those situations could help to minimize the impact of the disorder and improve the quality of life of patients with schizophrenia. Assessing the determinant factors of quality of life of patients with schizophrenia is particularly important for low-income countries like Ethiopia; for example, only few studies have examined the determinant factors of quality of life of patients attending psychiatry clinic follow-up service. Therefore, this study is aimed at assessing the quality of life and associated factors of schizophrenia patients attending follow-up treatment at the Jimma University Medical Center (JUMC), Southwest Ethiopia.

## 2. Methods

### 2.1. Study Setting

The study was conducted in JUMC, which is one of the oldest governmental hospitals established in 1937 during the Ethio-Italian war in the service of their soldiers. The psychiatric clinic of JUMC was established in 1996 and renders services including inpatient and outpatients for the about 15 million population as catchment in Southwest Ethiopia. Currently, there are more than 1000 patients who are attending follow-up treatments at the Outpatient Department (OPD) monthly, and on average, around 50 patients are visiting daily.

### 2.2. Study Design

The hospital-based cross-sectional study design was employed in Jimma University Medical Center, psychiatric clinic, Southwest Ethiopia.

### 2.3. Sampling Technique

The systematic random sampling technique was used to select study participants. The total number of schizophrenic patients attending follow-up was reviewed, and about 720 patients were attended follow-up treatment over two month periods prior of data collection. A sample fraction of two was used to select study participants. The first patient included in the study was determined by the lottery method, and every subsequent two patients were selected until the required sample size was reached (*n* = 352).

### 2.4. Sample Size

The minimum number of sample size required for this study was determined by using the formula to estimate the single population mean, *n* = (*Z* alpha/2)^2^(*δ*^2^)/*d*^2^, by using the following assumptions: standard deviation (SD) of the mean quality of life score 9.13 [24], a 95% confidence interval (CI) of 1.96 (*Z* alpha/2 = 1.96), a 1% margin of error (*d*, 0.01), and a nonresponse rate of 10%. We applied the single population mean formula to give *n* = (1.96)^2^∗(9.13)^2^/(1)^2^ = 320. Then, adding 10% of non-response rate (32), the final sample size was 352.

### 2.5. Study Procedures

Data was collected by a face to face interview by using semistructured and pretested questionnaires by four Master of Science in psychiatry students. Five-day training on the study objectives, data collection methods, ethical issues, and data collection tools was given for data collectors and the supervisors. A pretest was conducted on 5% of the sample at Agaro General Hospital, 45 km from Jimma. Data collectors were supervised by two Master of Science students in public health and the principal investigator. On each day of data collection, the completed questionnaires were checked for completeness. The collected data were entered into a computer and processed.

We assessed the patient's quality of life using the World Health Organization Quality of Life Scale–Brief version (WHOQOL–BREF) which is a 26-item self-administered generic questionnaire. It is a short version of the WHOQOL–100 scale [19]. The WHOQOL-BREF is a sound, cross-culturally valid assessment of QoL, as reflected by its four domains: physical, psychological, social, and environment [20]. It is suitable for the assessment of QoL in patients with schizophrenia [21]. It produces a profile with four domain scores: physical health (7 items), psychological health (6 items), social relationships (3 items), and environmental domain (8 items) as well as two separately scored items about the individuals' perception of their quality of life (QI) and health (Q2). The scores of items in each domain were added, and the mean was calculated to give the domain score. The reliability of WHOQOL-BREF in this study was 0.96 (Cronbach's alpha (*α*)).

We used a structured questionnaire to assess the following potential explanatory variables: socioeconomic factors (age, sex, ethnicity, religion, marital status, educational status, occupational status, and income) and the Morisky, Green, and Levine Medication Adherence Scale used to assess medication adherence. It consists of four items with a scoring scheme of yes = 1 and no = 0. The items in the Morisky, Green, and Levine Medication Adherence Scale were summed up to give a range of scores from zero to four (0-4). For this study, any participant who scored 1 or more was considered nonadherent while those who score 0 were taken as adherent [22]. The positive and negative syndrome scale (PANSS) was used to assess positive and negative symptoms of schizophrenia and severity of symptoms; it has 30 items which were rated on a 7-point Likert scale, and 7 are grouped to form a negative scale, 7 are grouped to form a positive scale, and 16 items constitute a general psychopathology scale. The Alcohol, Smoking, and Substance Involvement Screening Test (ASSIST) was used to assess substance use-related problems. It has 8 items.

### 2.6. Data Processing and Analysis

Collected data were checked, coded, and entered into EpiData version 3.1. Then, the data were exported to the Statistical Package for the Social Sciences version 21.0 for further analysis. Descriptive statistics, such as frequency, mean, and standard deviation, was computed, and bivariate and multivariable linear regression analyses were used to identify factors associated with the outcome variable. Factors associated with the outcome variables that had a *P* value < 0.25 in the bivariate analysis were included in the multivariable analysis. Multicollinearity, assumptions of linearity, and normality of the distribution were checked. Statistical significance was set at a *P* value < 0.05. Unstandardized beta (*β*) coefficients with 95% confidence interval (CI) were computed to assess the level of association and statistical significance.

### 2.7. Ethical Consideration

Ethical clearance was obtained from the Research, Ethical Review Board of Jimma University Institute of Health, and the study was performed according to the Declaration of Helsinki. Written informed consent was obtained from study participants. Participants were told the right to refuse or discontinue participation at any time they want and the chance to ask anything about the study. After data entry was completed, the questionnaires were kept securely locked.

## 3. Results

### 3.1. Sociodemographic Characteristics

A total of 351 patients with schizophrenia participated in the study. The response rate was 99.7%. The mean age of participants was 33 ± 8 years standard deviation (SD) and ranges from 18 to 54 years. More than half of the study participants were married; one-third was single (never married). The median score of the average monthly income of the participants was 600 Ethiopian Birr with an interquartile range of 1000 Ethiopian Birr (Table 1).

### 3.2. Quality of Life of Patients with Schizophrenia

The mean total quality of life score was found to be 74.34 ± 15.83. The highest QoL domain of the patients with schizophrenia in this study was the environmental health domain mean score, followed by the physical health domain, psychological health domain, and social relationship domain mean score. The WHOQOL-BREF scale demonstrated a high internal consistency reliability coefficient (Cronbach′s alpha = 0.96) (Table 2).

### 3.3. Clinically Related Characteristics

The median score of the duration of the illness of the participant was 4 years (interquartile range (IQR) 6). The median score of the duration of treatment was 4 years (IQR 4). The mean total score of PANSS in this study was 63.70 (SD ± 34.78). General psychopathology scored the highest score among the psychopathology. Two-third of the study participants were adherent to the prescribed medication. Regarding substance use, khat was the most commonly used substance in the last three months before the study (*n* = 248/351); 70.7% and about half of the study participants had used tobacco in the last three months prior to the study.

### 3.4. Factors Associated with Patient Quality of Life

Average monthly income, medication nonadherence, positive symptom, negative symptom, general psychopathology, having no history of admission, having a comorbid physical illness, ever use of tobacco, and ever use of khat were found to be statistically significantly associated with quality of life (Table 3).

The final multivariable linear regression model showed that participants' average monthly income (*β*: 5.81; 95% CI, 3.45-8.18), medication nonadherence (*β*: -5.81; 95% CI, -8.24-(-3.41)), positive symptom (*β*: -0.33; 95% CI, -0.49-(-0.17)), negative symptom (*β*: -0.26; 95% CI, -0.45-(-0.06)), general psychopathology (*β*: -0.22; 95% CI, -0.32-(-0.12)), ever use of tobacco (*β*: -3.95; 95% CI, -5.34-(-0.95)), ever use of khat (*β*: -3.95; 95% CI, -6.02-(-1.88)), and comorbid physical illness (*β*: -4.69; 95% CI, -8.50-(-0.88)) were found to be statistically significantly associated with quality of life. With a unit increase in the monthly income of the patient, the total quality of life score increased by 5.81 (*P* < 0.001). Being nonadherent to the prescribed medication decreases the total quality of life score by 5.82 (*P* < 0.001) as compared to those patients with schizophrenia who are adherent to their prescribed medications. As the mean score of positive symptom increased by one unit, the total quality of life score decreased by 0.33 (*P* < 0.001). As the mean score of negative symptom increased by one unit, the total quality of life score decreased by 0.26 (*P* = 0.009) (Table 4).

## 4. Discussion

This cross-sectional study identified factors associated with the quality of life of people with schizophrenia attending follow-up treatment at JUMC, Southwest Ethiopia. The mean total quality of life score was found to be 74.34 ± 15.83, and the domain with the lowest mean score is the social relationship domain (7.57 ± 2.36). The lowest mean score on the social relationship domain could be due to the negative symptoms present in these patients, which affect the patient's ability to live independently, to perform activities of daily living, and to be socially active and maintain personal relationships. Also, it could be due to the fact that patients with chronic mental illness like schizophrenia dislike the stigma of mental illness, which excludes them from social life [23].

In the current study, there was a significant positive correlation between the average monthly income of the patients with schizophrenia and their total QoL. Similar results about the relationship between income and QoL were also reported from different countries like Jordan and India [2, 12]. This was understandable as patients who were financially satisfied had expectations like normal people and able to meet their basic needs.

Participants who were nonadherent to the prescribed medications had a more likelihood of having a lower mean score of the total QoL scores than patients who were adherent to the prescribed medication. This finding was supported by the study done in Nigeria that showed medication nonadherence was negatively correlated with the well QoL scores [16]. This could be explained by the fact that being nonadherent to medications may lead to relapse, worsening of symptoms, and deterioration of the patients' mental health.

Furthermore, the result of this study indicated that there were negative relationships between QoL and the severity of the psychiatric symptoms. These results were in accordance with previously reported results in similar populations which reported that the severity of the illness affects the QoL of patients with schizophrenia [2, 12, 16, 17]. This could be due to the nature and chronic course of the illness.

Regarding substance use, patients who had a history of tobacco use in the last three months prior to the study had three times likelihood of decreased scores of the total quality of life as compared to nonusers; patients who had a history of khat use in the last three months prior to the study had four times likelihood of decreased scores of the total quality of life as compared to nonusers. This is in line with another study reporting that patients who are dually diagnosed with schizophrenia and substance use had significantly lower QoL scores than patients who did not use the substance [14]. This could be due to the fact that those patients with schizophrenia who have comorbid substance use problems have a poorer course of the illnesses, and it is more attributable to the direct effect of drugs on the worsening symptoms and the financial costs. Contrary to our results, dual-diagnosed patients with schizophrenia and substance use in the study from Australia [24] expressed higher levels of satisfaction with their QoL compared with noncomorbid patients. This inconsistency could be related to several factors, such as differences in the samples and differences in tools.

In summary, this study has substantial clinical implications for health services in improving the quality of life of people with schizophrenia. We used sound and cross-culturally valid data collection tools and incorporate several factors to reflect an actual representation of the quality of life of patients. In addition, the study was carried out with a maximal response rate. Recommendation was made for the concerned body on the way to develop strategies for community support programs to increase and enhance social relationships: creating income-generating activities for patients with schizophrenia, developing strategies to enhance public awareness and education, and encouraging psychosocial treatments like case management services and peer support programs in order to enhance the patients' capacity for building broader networks of relationships.

However, this study used a self-reported method to assess QoL which may lead to a lack of objective measures and an overreporting of QoL. Also, data on substance use in the previous 3 months were collected by an interview, which has a risk of recall bias. Another limitation is that data on comorbid physical illness was reviewed from the patient record which underestimated the case.

## 5. Conclusions

The domain of social relationship had the lowest mean score of the WHOQOL-BREF for patients with schizophrenia. Positive symptoms, negative symptoms, general psychopathologies, comorbid physical illness, tobacco use in the last three months prior to the study, khat use in the last three months prior to the study, and medication nonadherence were negatively associated with good QoL.

## Figures and Tables

**Table 1:** Sociodemographic characteristics of patients with schizophrenia attending follow-up clinic, JUMC, Southwest Ethiopia, June-July 2018 (*n* = 351).

Variables	Variable categories	Frequency (*n*)	Percentage (%)
Sex	Male	242	68.9
	Female	109	31.1

Marital status	Never married	120	34.2
	Married	193	55.0
	Divorced	30	8.5
	Widowed	8	2.3

Religion	Muslim	228	65.0
	Orthodox Christian	66	18.8
	Protestant Christian	57	16.2

Ethnicity	Oromo	236	67.2
	Amhara	38	10.8
	Yem	6	1.7
	Dawuro	13	3.7
	Kafa	30	8.5
	Other^∗^	28	8.0

Occupational status	Government worker	79	22.5
	Farmer	84	23.9
	Merchant	35	10.0
	Housewife	45	12.8
	Daily labor	45	12.8
	Other^∗∗^	63	17.9

Educational status	No formal education	95	27.1
	Primary school	99	28.2
	Secondary school	77	21.9
	Above secondary	80	22.8

Residence	Urban	203	57.8
	Rural	148	42.2

Other^∗^: Tigre, Walayta; other^∗∗^: student.

**Table 2:** Mean distribution of WHOQOL-BREF scale of patients with schizophrenia attending follow-up clinic, JUMC, Southwest Ethiopia, June-July 2018.

Variables	Mean (±SD)	Range	
		Minimum	Maximum
WHOQOL-BREF total	74.34 ± 15.83	32	107
Domains of WHOQOL-BREF			
Physical health	20.49 ± 4.25	9	29
Psychological health	18.15 ± 4.14	6	27
Social relationships	7.57 ± 2.36	3	15
Environmental health	22.42 ± 5.08	8	34
Item 1	2.79 ± 0.89	1	5
Item 2	2.87 ± 0.93	1	5

SD: standard deviation; item 1: overall perception of quality of life of respondents; item 2: general health satisfaction.

**Table 3:** Bivariate linear regression analysis of associated factors of quality of life of patients with schizophrenia attending follow-up clinic, JUMC, Southwest Ethiopia, June-July 2018.

Variable	*R* ^2^	*β*	95% CI	*P* value
Age of participants	0.001	0.002	-0.208-0.211	0.987
Female participants	0.009	3.225	-0.357-6.807	0.077
Participant's marital status				
Married^∗^	0.003	Reference value		
Single	0.006	-2.625	-6.124-0.873	0.141
Divorced	0.000	0.361	-5.593-6.316	0.905
Widowed	0.004	6.563	-4.570-17.697	0.247
Participant's educational status				
College or above^∗^	0.001	Reference value		
No education	0.002	-1.688	-5.430-2.055	0.376
Primary education	0.005	2.388	-1.303-6.079	0.204
Secondary education	0.000	0.451	-3.571-4.474	0.826
Participant's occupational status	0.000	Reference value		
Government^∗^	0.002	-1.741	-5.638-2.157	0.380
Private	0.000	0.071	-5.485-5.627	0.980
Unemployed	0.000	0.761	-4.217-5.740	0.764
Monthly income	0.150	15.998	11.995-20.000	<0.001
Duration of illness	0.004	-2.850	-7.718-2.018	0.250
Duration of treatment	0.003	-0.193	-0.539-0.154	0.275
Positive symptom	0.572	-1.289	-1.407-(-1.172)	<0.001
Negative symptom	0.628	-1.317	-1.424-(-1.210)	<0.001
General psychopathology	0.620	-0.688	-0.744-(-0.631)	<0.001
Medication nonadherence	0.427	-21.061	-23.627-(-18.494)	<0.001
No history of admission	0.040	6.385	3.087-9.682	<0.001
Comorbid physical illness	0.080	-19.338	-26.225-(-12.451)	<0.001
Ever use of tobacco	0.321	-18.069	-20.838-(-15.299)	<0.001
Ever use of khat	0.122	-12.111	-15.537-(-8.684)	<0.001
Ever use of alcohol	0.003	1.829	-1.889-5.547	0.334

^∗^Reference group; other: housewife and student.

**Table 4:** Multivariable linear regression analysis for independent predictors of quality of life of patients with schizophrenia attending follow-up clinic, JUMC, Southwest Ethiopia, June-July 2018.

	Unstandardized coefficient *β*	*P* value at 95% CI	95% confidence interval for *β*	
			Lower	Upper
Constant	74.23	≥0.001	71.06	87.39
Monthly income	5.81	≥0.001	3.45	8.18
Medication nonadherence	-5.82	≥0.001	-8.24	-3.41
Positive symptom	-0.33	0.003	-0.49	-0.17
Negative symptom	-0.26	0.009	-0.45	-0.06
General psychopathology	-0.22	≥0.001	-0.32	-0.12
Comorbid physical illness	-4.69	0.016	-8.50	-0.88
Ever use of tobacco	-3.15	0.005	-5.34	-0.95
Ever use of khat	-3.95	≥0.001	-6.02	-1.88

*R* = 0.872, *R*^2^ = 0.760, *F* = 82.058, and *P* < 0.001.

## Data Availability

The datasets generated during the current study are available on reasonable request from the corresponding author.

## Abbreviations

CI: Confidence interval
IQR: Interquartile range
JUMC: Jimma University Medical Center
PANSS: Positive and negative syndrome scale
QoL: Quality of life
SD: Standard deviation
WHOQOL-BREF: World Health Organization Quality of Life Scale–Brief version.
